# Severe rhabdomyolysis associated with severe fever with thrombocytopenia syndrome in a married couple: a case report

**DOI:** 10.1186/s12879-019-4535-9

**Published:** 2019-10-24

**Authors:** Osamu Imataki, Makiko Uemura, Hisashi Masugata

**Affiliations:** 1 Division of Hematology, Department of Internal Medicine, Faculty of Medicine, Kagawa University, 1750-1 Ikenobe, Miki-town, Kita-county, Kagawa 761-0793 Japan; 2 Department of Integrated Medicine, Faculty of Medicine, Kagawa University, Miki, Kagawa Japan

**Keywords:** Severe fever with thrombocytopenia syndrome (SFTS), SFTS virus (SFTSV), Rhabdomyolysis, Immune response

## Abstract

**Background:**

Severe fever with thrombocytopenia syndrome (SFTS) is a tick-borne infection that has recently emerged. This infectious disease is due to the transfer of SFTS virus (SFTSV) from the infected blood of animals to humans. Approximately 30% of patients who are infected with SFTS die from multiorgan failure associated with severe infection, systemic inflammatory response syndrome, or disseminated intravascular coagulation. We treated an elderly Japanese couple (husband and wife) who had genetically identical SFTSV infections and who both developed severe rhabdomyolysis.

**Case presentation:**

An 80-year-old man presented to the clinic with a fever; his 74-year-old wife presented with a fever 9 days later. Their laboratory results at diagnosis showed severe rhabdomyolysis with significantly elevated creatinine kinase (detected levels: husband, 9546 U/L; wife, 15,933 U/L). The creatinine kinase isozyme was 100% MM type in both patients. In both the husband and wife, SFTSV was identified with real-time polymerase chain reaction analysis. The detected SFTSVs in both the husband and wife were identical according to the genome sequence analysis. The husband’s bone marrow indicated macrophage activation syndrome, but he responded to supportive therapy. He was discharged after being hospitalized for 32 days. The wife was admitted to our hospital in critical condition 2 days after SFTS symptom onset. She died of multiorgan failure 8 days after onset, despite being cared for in an intensive care unit. Both of the patients presented with rhabdomyolysis following SFTS symptom onset. The patients’ clinical outcomes were different from each other; i.e., the husband survived, and the wife died.

**Conclusions:**

SFTSV infection-associated rhabdomyolysis has been reported in one patient, and simultaneous onset in two related patients has not been described previously. Our findings suggest that similar biological responses occurred, but they resulted in different clinical outcomes in the patients infected by the identical SFTSV isolates. Notably, a patient’s clinical outcome depends on their own immune response. We suggest that one component of viral rhabdomyolysis involves immune-mediated responses. Severe immunological responses may adversely affect the treatment outcome, as demonstrated by the wife’s clinical course. Our findings demonstrate that a patient’s immune response contributes to their prognosis following SFTSV infection.

## Background

Severe fever with thrombocytopenia syndrome (SFTS) is a novel infectious disease that was first reported in China in 2011 and has been increasingly reported since [1]. SFTS is a disease caused by the SFTS virus (SFTSV), a novel bunyavirus that can spread from wildlife to people through tick bites [2]. Multiorgan failure has been reported in severe cases, and mortality rates of up to 30% have been reported. SFTS is a tick-borne blood infection, and most patients show evidence or symptoms suggestive of tick bites [1]. Unexpectedly, human-to-human transmission was reported in 2011 [3]. This case critically impacted nosocomial infection precautions among health care workers [4]. Endemically infected populations have been described in isolated communities, suggesting the possibility of human-to-human transmission [3, 5, 6]. In the case of human-to-human transmission, bodily fluids contaminated with blood are the primary transmitters of virus particles.

We treated a husband and wife who acquired an SFTSV infection at their home. The aim of this case report is to report a new scenario regarding SFTS. These cases indicated in-house familial transmission of SFTSV, probably through direct blood-to-blood contact [3, 5]. These cases confirm the possibility that a single genetically identical type of SFTSV can lead to different SFTS outcomes in different patients, indicating that SFTSV virulence varies according to the host’s immune status.

## Case presentations

### Husband

**Fig. 1:**
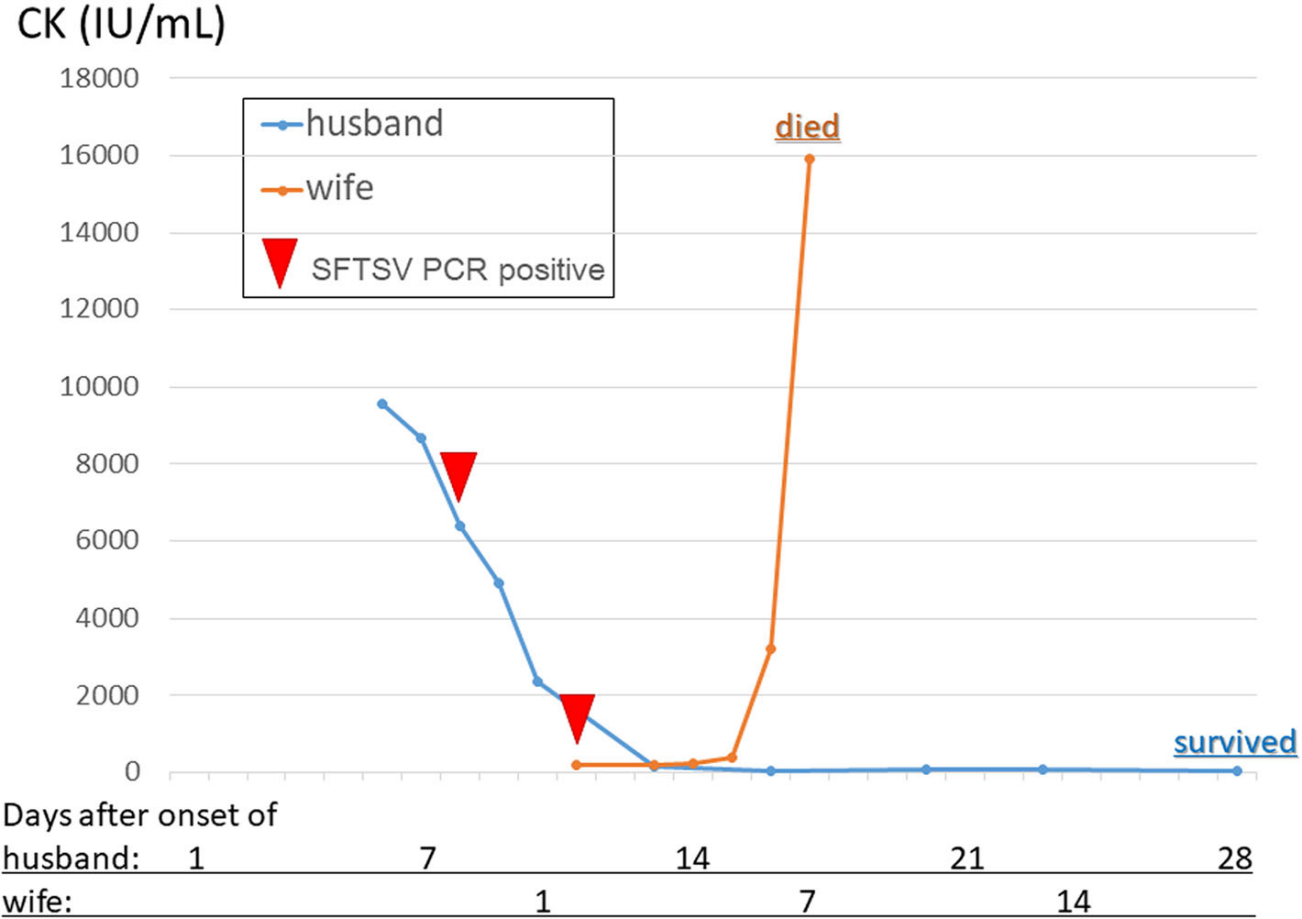
Clinical courses of the husband and wife. The figure shows the elevated creatinine kinase (CK) levels in both patients. The husband’s CK level peaked at symptom onset and decreased gradually after admission to the hospital. The CK levels in the wife increased dramatically 6 days after admission, and she died within 1 week of admission. (SFTSV, severe fever with thrombocytopenia syndrome virus)

The patient was an 80-year-old Japanese man who presented with a fever of 38.0 °C. His performance status was 4, but he did not complain of muscle pain symptoms. His medical history showed that he had an appendiceal resection at 20 years of age, a thyroid tumor resection at 77 years of age, and a left inguinal hernia operation at 78 years of age. Table 1 shows his laboratory data after admittance to the hospital. His creatinine kinase level was significantly elevated, and isozyme analysis revealed a 100% MM isotype. We initiated antimicrobial therapy with meropenem, as *Streptococcus oralis* was detected in his blood culture. The strain of bacteria was susceptible to meropenem, and antimicrobial therapy was discontinued on day 6 after fever resolution. Computed tomography revealed no enlarged lymph nodes (all lymph nodes were < 15 mm in diameter) and no organic lesions. Bone marrow aspirates indicated reactive granulocytosis with increased plasmacytes (4.6%) and active phagocytosis by macrophages (0.6%). A karyotype analysis showed that there were 46 XY chromosomes (normal karyotype) in all 20 cells that were analyzed. The patient was diagnosed with SFTSV by polymerase chain reaction (PCR) analysis. After the confirmation of SFTSV infection, the patient received supportive therapy including antimicrobials, a platelet transfusion, and rehydration. Eight days after symptom onset, his fever was resolved, and he was discharged after 32 days of hospitalization (Fig. 1).

**Table 1:** Husband’s data

【CBC】		
WBC	750	/mL
RBC	471 × 10^4^	/mL
HGB	14.8	g/dL
HCT	40.2	%
PLT	3.7 × 10^4^	/mL
【leukocyte classification】		
STAB	9.0	%
SEG	52.0	%
EOS	0.0	%
BASO	0.0	%
LYM	32.0	%
MONO	7.0	%
Erythroblast	1	/100
Ret	2.91 × 10^4^	/mL
【coagulation】		
PT	85	%
PT-INR	1.08	
APTT	41.7	sec
Fibrinogen	240	mg/dL
ATIII	93	%
D-dimer	4.1	mg/mL
FDP	11.4	mg/mL
【biochemistry】		
CRP	0.02	mg/dl
TP	6.0	g/dl
ALB	3.4	g/dl
BUN	15.3	mg/dl
Cr	0.71	mg/dl
T-Bil	0.7	mg/dl
GOT	303	U/L
GPT	83	U/L
ALP	225	U/L
LDH	936	U/L
gGTP	73	U/L
NA	132	mmol/L
K	3.7	mmol/L
CL	100	mmol/L
CA	7.3	mg/dL
CK	9516	U/L
ferritin	4771.0	ng/mL

### Wife

The patient was a 74-year-old Japanese woman whose symptoms appeared 9 days after her husband’s symptoms appeared. She had no reported medical history. She was diagnosed with SFTS by PCR analysis. The SFTSV detected in the wife was genetically identical to that detected in the husband by genome sequence analysis. Her creatinine kinase levels increased dramatically from 206 mg/dL to 15,933 mg/dL within 6 days of presentation. Table 2 shows her laboratory findings at symptom onset. Despite treatment in our intensive care unit, she died from multiorgan failure 8 days after presenting to the clinic (Fig. 1). The wife did not undergo bone marrow aspiration.

**Table 2:** Wife’s data

【CBC】		
WBC	5000	/mL
RBC	448 × 10^4^	/mL
HGB	13.7	g/dL
HCT	39.3	%
PLT	15.4 × 10^4^	/mL
【leukocyte classification】		
Neut	70.0	%
EOS	0.4	%
BASO	0.4	%
LYM	18.0	%
MONO	11.2	%
Ret	2.66 × 10^4^	/mL
【coagulation】		
PT	58	%
PT-INR	1.35	
APTT	27.3	sec
Fibrinogen	458	mg/dL
ATIII	93	%
D-dimer	4.1	mg/mL
FDP	11.4	mg/mL
【biocheistry】		
CRP	2.73	mg/dl
TP	8.0	g/dl
ALB	3.7	g/dl
BUN	12.5	mg/dl
Cr	0.91	mg/dl
T-Bil	0.8	mg/dl
GOT	124	U/L
GPT	73	U/L
ALP	255	U/L
LDH	313	U/L
gGTP	160	U/L
NA	132	mmol/L
K	4.1	mmol/L
CL	98	mmol/L
CA	9.1	mg/dL
CK	206	U/L

The SFTSV genomes of both patients were identical, as indicated by direct sequencing; this genetic identity was reflected in the rhabdomyolysis experienced by both patients. However, the two patients’ clinical outcomes were different; i.e., the husband survived following the spontaneous regression of rhabdomyolysis, and the wife died of multiorgan failure and systemic inflammatory response syndrome secondary to severe rhabdomyolysis. This difference suggests that patient factors, that is, immune system function, contributed to the SFTS prognosis in each individual.

## Discussion and conclusions

Within a community or a family, the transmission of SFTSV usually occurs through direct blood transmission [7, 8]. However, person-to-person blood-borne transmission, as likely occurred in our patients, has been reported in some studies [4, 5, 9]. The only confirmed prognostic factor for SFTS is the presence of comorbidities, such as heart failure, diabetes mellitus, or other organ dysfunctions [10]. Our couple was infected with genetically identical viruses, but the patients’ biological responses and clinical outcomes differed. One explanation is the differences in the patients’ immunological responses to SFTSV. Although rhabdomyolysis occurred in both patients, their clinical outcomes were very different. The husband gradually recovered, but the wife’s condition worsened, and she died. In most patients with virus-associated rhabdomyolysis, biopsy does not reveal specific findings [11], and treatment is therefore supportive and short-term. In addition, time is required for a natural decrease in the viral load and the spontaneous resolution of muscle damage in most patients [11]. Based on the findings in our two patients, future studies should evaluate patient factors associated with responses to and protection from SFTSV infection. For instance, excessive immune responses to SFTSV might be suppressed using corticosteroids.

Myolysis-complicated SFTS has been reported [12] in only one case report. Rhabdomyolysis comprehensively affects skeletal muscle and manifests as elevated serum creatinine kinase levels. Rhabdomyolysis is a systemic disease that is not accompanied by the focal manifestation of abscesses [13]. However, the condition is sometimes complicated by an additional systemic infection [13]. The pathogenesis of infectious rhabdomyolysis is considered to be associated with local or systemic metabolic changes that are related to local or systemic infections [14], but the mechanisms underlying the pathogenesis have not been fully elucidated. A variety of viruses may cause rhabdomyolysis [11, 15, 16], with influenza A and B viruses being the most common causes [16]. One potential pathophysiological mechanism of viral rhabdomyolysis may be the direct infection of muscles by the virus, followed by associated immune-mediated responses and resulting metabolic damage. A second possible pathophysiological mechanism may be variable host immune responses among patients. The immunological response theory includes immunological cell infiltration and cytokine release. The third potential pathophysiological mechanism suggests that all of the symptoms are simply secondary to fever. Clinically, we often observe a relation between fever spikes and subsequent increases in serum creatinine kinase levels [16]. However, it is important to eliminate the possibility of drug-induced rhabdomyolysis. In most patients, myositis is usually self-limiting; however, our female patient’s condition worsened due to systemic immunological response syndrome. Virus-associated rhabdomyolysis may reveal an underlying structural or metabolic myopathy [17], indicating systemic metabolic disorders local or physiological changes. Virus-associated rhabdomyolysis has been reported in aggregated case series and reviews [18, 19], and these authors have discussed potential underlying biological mechanisms.

In conclusion, we reported the cases of a husband and wife who were both infected with SFTSV in their home and developed virus-associated rhabdomyolysis. Both patients showed similar biological responses to the genetically identical SFTSVs. Our findings suggest that a patient’s immune response contributes to the disease course following SFTSV infection. Our results may help with considerations for treatment for intrafamilial SFTS transmission.

## Data Availability

No other data were analyzed in this study.

## Abbreviations

SFTS: Severe fever with thrombocytopenia syndrome
SFTSV: Severe fever with thrombocytopenia syndrome virus
